# Beta-Actin Is Required for Proper Mouse Neural Crest Ontogeny

**DOI:** 10.1371/journal.pone.0085608

**Published:** 2014-01-07

**Authors:** Davina Tondeleir, Rivka Noelanders, Karima Bakkali, Christophe Ampe

**Affiliations:** Department of Biochemistry, Faculty of Medicine and Health Sciences, Ghent University, Ghent, Belgium; University of Colorado, Boulder, United States of America

## Abstract

The mouse genome consists of six functional actin genes of which the expression patterns are temporally and spatially regulated during development and in the adult organism. Deletion of beta-actin in mouse is lethal during embryonic development, although there is compensatory expression of other actin isoforms. This suggests different isoform specific functions and, more in particular, an important function for beta-actin during early mammalian development. We here report a role for beta-actin during neural crest ontogeny. Although beta-actin null neural crest cells show expression of neural crest markers, less cells delaminate and their migration arrests shortly after. These phenotypes were associated with elevated apoptosis levels in neural crest cells, whereas proliferation levels were unchanged. Specifically the pre-migratory neural crest cells displayed higher levels of apoptosis, suggesting increased apoptosis in the neural tube accounts for the decreased amount of migrating neural crest cells seen in the beta-actin null embryos. These cells additionally displayed a lack of membrane bound N-cadherin and dramatic decrease in cadherin-11 expression which was more pronounced in the pre-migratory neural crest population, potentially indicating linkage between the cadherin-11 expression and apoptosis. By inhibiting ROCK ex vivo, the knockout neural crest cells regained migratory capacity and cadherin-11 expression was upregulated. We conclude that the presence of beta-actin is vital for survival, specifically of pre-migratory neural crest cells, their proper emigration from the neural tube and their subsequent migration. Furthermore, the absence of beta-actin affects cadherin-11 and N-cadherin function, which could partly be alleviated by ROCK inhibition, situating the Rho-ROCK signaling in a feedback loop with cadherin-11.

## Introduction

Actins are highly conserved proteins throughout evolution [1]. The human genome consists of six functional actin genes and more than twenty pseudogenes [2]. Also other mammals including mouse encode six functional paralogs (Tondeleir et al., in preparation). The expression patterns are temporally and spatially regulated during development and in the adult organism, suggesting different isoform specific functions [1], [3], [4].

Beta-actin appears to be the only actin isoform that is targeted to specific cellular compartments via a specific region in its 3′UTR region, called the zipcode, and this has been correlated with migration and directional growth cone motility [5], [6], [7], [8]. Hence these subcellular differences in beta-actin protein level could play an important role in neuronal cells. This was recently addressed for beta-actin in motor neurons and in the mammalian central nervous system using a central nervous system specific knockout mouse [9]. In the surviving adult mice, abnormalities were detected in hippocampus and cerebellum as well as localized defects in axonal crossing of the corpus callosum, indicating the importance of beta-actin for neuronal development.

We recently reported that ablation of beta-actin in mouse embryonic fibroblasts (MEFs) results in decreased migration capacity [10]. Given its role in cell migration this phenotype could be expected but the picture is more complex since the beta-actin knockout MEFs exhibited a genetic reprogramming that manifested itself mainly by actin isoform switching, increased TGFβ production and Rho-ROCK signaling. Interestingly, inhibiting the latter pathway restored migration of beta-actin knockout MEFs indicating that altered migration did not result from lack of actin polymerization capacity but rather from the combination of a changed genetic program in conjunction with altered signaling, thereby implicating beta-actin in signaling to gene expression regulation. Indeed, shortly after, we reported a role for beta-actin in nuclear signaling. Employing a beta-actin knockout mouse that was previously generated [11], we demonstrated that homozygous beta-actin knockout mice (Actb^−/−^) are lethal at stage E10.5 due to impaired primitive erythropoiesis leading to hypoxia [12].

In order to further contribute to the knowledge on the functions of beta-actin during mouse development, we explored in the beta-actin null embryos [11] whether specific cell populations were affected. We focused on the peripheral nervous system since this is fully expanding in mouse embryos at the time of lethality in beta-actin null embryos. To a large extent, this system specifically originates from the transient neural crest cell population [13], [14], [15]. This embryonic population arises in the developing central nervous system at the interface between the neural plate and the adjacent non-neural ectoderm. In addition to formation of the peripheral nervous system, the neural crest cells give rise to a plethora of derivatives including pigment cells, a major part of the cartilage and bone of the craniofacial structures, endocrine cells, cardiac structures, smooth muscle cells and tendons. To realize these various tasks the neural crest must migrate long distances and transform multiple times from one tissue related phenotype to another. The regulation of this tissue formation and cell sorting requires, amongst others, the presence of cadherins [16], [17], [18]. A phenomenon common to many species is the exchange of a type I cadherin for a type II cadherin during neural crest emigration [19], [20], [21]. However, such a cadherin switch in mouse is poorly documented.

While the peripheral nervous system is indeed in full expansion in wild type (Actb^+/+^) embryos, we observed a partial block in neural crest ontogeny in beta-actin null embryos (Actb^−/−^). Apoptosis levels in pre-migratory neural crest cells are doubled compared to Actb^+/+^ neural crest cells leading to a decreased number of migratory neural crest cells and identifying beta-actin as a vital protein for their survival, specifically in the early stages of development. Actb^−/−^ migratory neural crest cells show impaired migration behavior in addition to a dramatic decrease in cadherin-11 expression. These cells also lost N-cadherin expression in regions of cell-cell contact. Ex vivo, the migration defect could be restored by inhibiting the Rho kinase ROCK, as assessed in neural crest explants. ROCK inhibition also led to an increase in cadherin-11 levels in Actb^−/−^ neural crest cells. In summary, beta-actin proves essential for survival of pre-migratory neural crest cells, their proper emigration and subsequent migration. Beta-actin is also involved in cadherin function and cadherin-11 could potentially be involved in Rho-ROCK signaling.

## Materials and Methods

### Ethics Statement

The animal ethics committee of Ghent University approved all experiments performed on mice, approval number ECD10/29.

### Mice

The generation of the full heterozygous beta-actin knockout mice (Actb^+/−^) has been previously described [11]. Actb^+/−^ mice were crossed to generate control Actb^+/+^ embryos and Actb^−/−^ embryos. Mice were kept on an inbred BALB/c background.

### Antibodies & Riboprobes

Antibodies used in this study are mouse anti-neurofilament mAb (clone 2H3) from Developmental Studies Hybridoma Bank; mouse anti-beta-III tubulin mAb (clone5G8) from Promega; anti-digoxigenin-AP Ab from Roche; mouse anti-p75 NGF receptor mAb, rabbit anti-p75 NGF receptor pAb, mouse anti-Histone H3 (phospho S10) mAb, rabbit anti-vimentin mAb, all from Abcam; rat anti-E-cadherin mAb (clone DECMA-1) from Sigma-Aldrich; mouse anti-N-cadherin mAb (clone 3B9), mouse anti-cadherin-11 mAb (clone 5B2H5), anti-mouse Alexa Fluor 488, anti-rabbit Alexa Fluor 488, anti-rabbit Alexa Fluor 594, anti-rat Alexa Fluor 594, anti-mouse Alexa Fluor 647, anti-rabbit Alexa Fluor 647 all from Invitrogen. Sox10, Crabp1 and Pax3 riboprobes were kind gifts from Paul Trainor, Stowers Institute, Kansas City, US.

### Trunk Neural Tube Explant Assay

The method used to establish the neural crest outgrowth cultures were adapted from those described by Murphy et al. [22]. Briefly, for trunk neural tube explant cultures we used the neural tube regions corresponding to the last formed 8 somite pairs of E9.0 mouse embryos. Neural tubes were isolated from the embryo by incubating them for 20 min in dispase I (Roche, 2,4 U/ml) at room temperature. Neural tubes were subsequently plated on plastic dishes or glass slides coated with 10–20 µg/ml fibronectin and grown for 48 hours in 5% CO_2_ and 37°C in Dulbecco’s modified Eagle medium supplemented with 10% fetal bovine serum. For experiments with ROCK inhibition, 20 µM of ROCK inhibitor was added to the medium (Calbiochem, Merck KGaA). Quantification of neural tube outgrowths was done as previously described [23]. Briefly, a migration index (MI) was obtained by taking the area of the entire explant and subtracting from it the area of the dense central neural tube mass. We controlled for differences in migration area resulting from random variations in the shape of the explant by dividing the outgrowth area (mm2) by the perimeter (mm) of the explant. This normalized number (in mm) is referred to as the migration index and provides an estimate of the migration rate of the neural crest cells.

### Paraffin Histology

Dissected samples were fixed overnight in 4% paraformaldehyde (PFA) in phosphate buffered saline (PBS) at 4°C, dehydrated through ethanol series before incubation in xyleen and embedding in paraffin. Embryos were sectioned at 6 µm and subsequently used for immunofluorescence or in situ hybridization.

### TUNEL Assay

TUNEL assays were performed using the In situ Cell Detection Kit, Fluorescein from Roche, according to the manufacturer’s instructions. For the combined TUNEL assay with immunohistochemistry using the rabbit anti-p75 nerve growth factor receptor pAb, the TUNEL assay was performed after the immunohistochemistry protocol, starting from the blocking step.

### Immunohistochemistry

Immunohistochemistry on whole mount embryos was performed as previously described [24]. Briefly, embryos were fixed in MeOH:DMSO (4∶1) overnight at 4°C, treated with MeOH:DMSO:H_2_O_2_ (4∶1∶1) for 5–10 hours at room temperature to block endogenous peroxidase activity and stored in methanol at −20°C. The embryos were subsequently rehydrated 50% MeOH in PBS (PBSMT) and incubated with the primary antibody in PBSMT overnight at 4°C. Following washes in PBSMT for 5 hours at room temperature, embryos were incubated with the ABC reagent, in PBSMT overnight at 4°C. Following washes in PBSMT for 5 hours at room temperature and brief washes with PBT, the embryos were developed with 3.3-diaminobenzidine tetrahydrochloride (DAB) (Vector laboratories). The reaction was stopped by fixing the embryos in 4% PFA in PBS at room temperature for 1 hour.

### Immunofluorescence

For immunofluorescence on neural tube explants, the neural tube was first removed from the dish or slide. Remaining cells were fixed with 4% paraformaldehyde in PHEM buffer pH6.9 (60 mM PIPES, 25 mM HEPES, 10 mM EGTA and 2 mM MgCl_2_) for 20 min at room temperature, permeabilized with 0.5% Triton X-100 in PBS for 15 min at room temperature and blocked with 2% bovine serum albumin/1% glycine in PBS for 30 min at room temperature. Cells were incubated with primary antibody in blocking solution for 45 min at 37°C. Following three washes with PBS of each 15 min at room temperature, cells were incubated with secondary antibody for 30 min at 37°C. Following three washes with PBS of each 15 min at room temperature, nuclei of cells were stained with DAPI (Sigma-Aldrich) and stored in PBS (dishes) or mounted with DABCO (Sigma) (slides). For immunofluorescence on embryo sections only trunk levels were used for analysis. Immunofluorescence on paraffin sections started by deparaffinization through ethanol series. Sections were microwaved in 0,01 M citrate buffer (pH 6.0) for 15 min at full power and washed in PBS. Sections were blocked in 10% goat serum/1% BSA in PBS for 1 hour at room temperature and incubated with primary antibody in blocking solution overnight at 4°C. Following four washes with PBS of each 30 min at room temperature, sections were incubated with secondary antibody for 2 hours at room temperature. Following four washes with PBS of each 15 min at room temperature, nuclei of cells were stained with DAPI and sections were mounted with DABCO mounting medium (Sigma-Aldrich). Quantification of immunofluorescence was done with Fluoview FV1000 software of Olympus or with Image J. Intensities of each chromophore were measured in the region immediately next to the neural tube (region A) or specifically in the dorsal part of the neural tube (region B). Pearson’s coefficients were measured for the p75 marker in combination with N-cadherin or with cadherin-11 and indicated the amount of colocalization between the markers.

### In situ Hybridization

Whole mount in situ hybridization was carried out as described by Miyoshi et al. [25]. Section in situ hybridization was done as described by Nagy [24].

### Gel Electrophoresis and Western Blot

**Figure 1:**
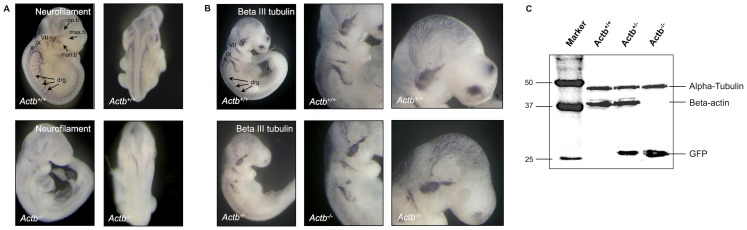
Actb^−/−^ embryos display abnormal peripheral nervous system at E10.25. (A) Neurofilament expression in Actb^+/+^ and Actb^−/−^ embryos. Aberrations of neurofilament expression in Actb^−/−^ embryos are visible at the level of all nerves. (B) Beta-III tubulin expression in Actb^+/+^ and Actb^−/−^ embryos. Aberrations of beta-III tubulin expression in Actb^−/−^ embryos are visible at the level of IX and X nerves and the dorsal root ganglia. (C) Western Blot analysis showing presence of GFP expression (driven by the endogenous promotor) [11] and the lack of beta-actin expression in Actb^−/−^ embryos. V: trigeminal nerve; VII: facial nerve; IX: glossopharyngeal nerve; X: vagus nerve; max.b: maxillary branch; man.b: mandibular branch; op.b: ophthalmic branch; drg: dorsal root ganglia. Panels depicting Actb^+/+^ and Actb^−/−^ embryos are of the same magnification.

Total embryonic lysates were prepared in 7 M urea, 2 M thio-urea, 0.5% TritonX-100, 40 mM dithiothreitol and protease inhibitors (1 µg/ml Leupeptin, 1 µg/ml Antipain, 1 µg/ml Aprotinin, 1.6 µg/ml Benzamidine). 8 µg of protein was loaded on 10% polyacrylamide gels. The beta-actin expression level was analyzed by western blotting using the appropriate set (see Figure 1) of primary and secondary antibodies.

### Imaging

Whole mount embryos were imaged on a Leica MS5 (Leica Microsystems) stereomicroscope. Digital images were acquired using a Leica camera. In situ hybridization sections were imaged using a SNAP-COOL camera (Roper Scientific) mounted on an Olympus Bx51 microscope (Olympus), with Plan Olympus 20x/0.40 or 40x/0.65 lens and RSImage Version 1.9.2 software (Roper Scientific). Neural tube explants and section immunofluorescence were imaged using an Olympus inverted XI71 microscope or an Olympus IX81 confocal microscope. Images were recorded using AnalySIS software (Olympus XI71) or using Fluoview FV1000 software (Olympus IX81).

### Statistical Analysis

Data were expressed as mean plus or minus SEM. Comparison between 2 data groups was done by the 2-sided Student t test. Results were quantified after at least three independent experiments. For statistical analysis, between 5 and 17 biological replicates were used in each condition for each genotype. Fluorescence intensities were normalized with the corresponding area of measurement.

## Results

### Loss of Beta-actin Affects Proper Neural Development

Given the severe ex vivo migration defects observed for Actb^−/−^ MEFs [10], we examined the possibility that specific embryonic cell populations also displayed migration defects at the time of organogenesis and therefore phenotyped Actb^−/−^ embryos. One of the more striking phenotypes is evident from immunohistochemistry of neurofilament protein. This protein is an intermediate filament specifically found in the axons of neurons of the central and peripheral nervous system [26]. At E10.25, the three branches of the trigeminal ganglion, the glossopharyngeal nerve and a large part of the facial nerve could not be observed in Actb^−/−^ embryos (Figure 1A). The vagus nerve is only slightly present on the backside of the embryo and dorsal root ganglia remain absent in Actb^−/−^ embryos, indicating that they lack a well-organized peripheral nervous system. To study this aspect further, we tested beta-III tubulin expression, which is widely regarded as neuronal marker [27]. The trigeminal ganglion of Actb^−/−^ embryos is hypomorphic and formation of the glossopharyngeal and the vagus nerves and the dorsal root ganglia are largely impaired (Figure 1B). The peripheral nervous system originates to a large extent from the transient neural crest cell population [13], [14], [15]. This prompted us to study this population and whether ontogeny problems of the neural crest cells are the underlying reason for the malformation of the peripheral nervous system. Interestingly, the heterozygous embryos did not display an intermediate phenotype, but resembled wild type embryos (data not shown), consistent with the high remaining beta-actin expression level (Figure 1C) [10]. Although Actb^−/−^ embryos displayed slight variations in size, somite numbers were comparable between Actb^+/+^ and Actb^−/−^ embryos, indicating that developmental delay is not causing the size variations.

### Actb^−/−^ Embryos have Less Migratory Neural Crest Cells

**Figure 2:**
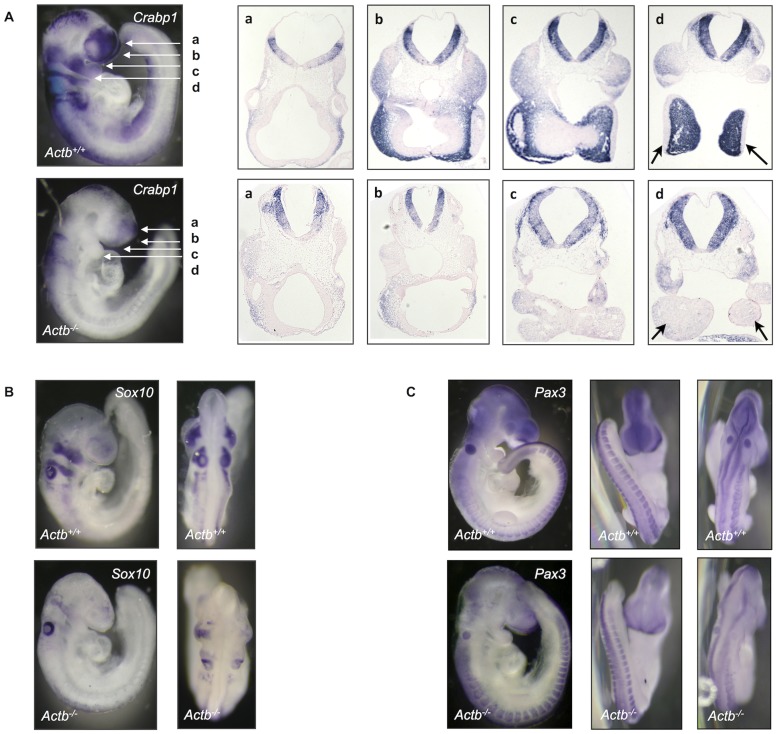
At E9.5 Actb^−/−^ embryos show defects in neural crest ontogeny. (A) Crabp1 whole mount and section ISH (at the indicated planes: a, b, c and d) on Actb^+/+^ and Actb^−/−^ embryos. For the latter all sections show absence of neural crest cells in certain regions of the sections. Actb^−/−^ neural crest cells are not able to reach the brachial arches (arrows in section d). (B) Sox10 whole mount embryo ISH marks the migrating neural crest. Sox10 ISH shows comparable results as the Crabp1 probe. (C) Pax3 marks the dermomyotome and the cranial neural crest. Compared to Actb^+/+^ embryos, the Actb^−/−^ embryos show a similar expression pattern of pax3 at the level of the dermomyotomes, but a reduction in the cranial region. Panels depicting Actb^+/+^ and Actb^−/−^ embryos are of the same magnification.

To analyze the distribution of neural crest cells in the Actb^−/−^ embryos, we first tested Crabp1 mRNA expression by in situ hybridization (ISH). Crabp1 is expressed both by neural crest cells in the dorsal neuroepithelium and by migratory neural crest cells [28]. The E9.5 Actb^−/−^ embryos show no neural crest cells in regions distant from the neural tube (Figure 2A). Detail images from section ISH show expression of Crabp1 in the neural tube of both Actb^+/+^ and Actb^−/−^ embryos, but significantly less migrating neural crest cells seem present in Actb^−/−^ embryos compared to Actb^+/+^ embryos. In wild type Actb^+/+^ embryos, neural crest cells already populate the first branchial arches at E9.5, while this does not occur in the Actb^−/−^ embryos (arrows in figure 2A, panel d). To confirm these results, we performed whole mount ISH with sox10 and pax3 probes. Sox10 marks the cranial neural crest cells that emigrate from the neural tube [29], [30]. Sox10 ISH at E9.5 indeed corroborates the aberrant Crabp1 staining pattern in Actb^−/−^ embryos (Figure 2B). Pax3 is expressed during early cranial neurogenesis and in the dermomyotome component of the somites [31], [32], [33]. While the expression of pax3 remained identical between Actb^+/+^ and Actb^−/−^ embryos in the trunk region, the cranial region of the Actb^+/+^ embryos displayed less intense pax3 coloring. Both observations indicate the presence of less migratory neural crest cells (Figure 2C).

### Actb^−/−^ Neural Crest Cells Display Increased Apoptosis

**Figure 3:**
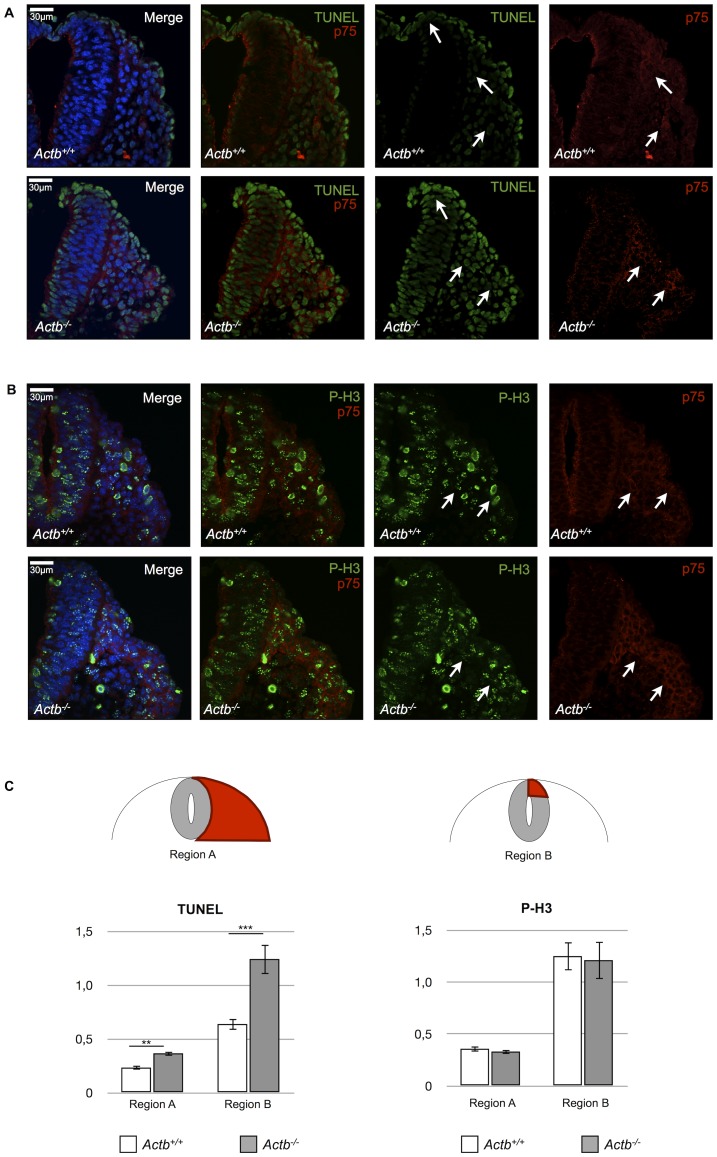
Increased apoptosis in Actb^−/−^ neural crest cells in vivo. (A) TUNEL analysis on Actb^+/+^ and Actb^−/−^ embryo sections stained with p75 at E9.5. Actb^−/−^ sections show increased apoptosis, both in the dorsal neural tube and migratory neural crest cells (indicated by white arrows) compared to Actb^+/+^ sections. (B) Phospho-Histone H3 immunohistochemistry on embryo sections stained with p75 at E9.5. Both Actb^+/+^ and Actb^−/−^ sections show similar staining pattern (indicated by white arrows). In (A) and (B) DAPI was used to reveal nuclear staining. (C) Quantification of TUNEL and phospho-Histone H3 intensities in migratory neural crest cells adjacent to the neural tube (region A) and in the dorsal neural tube (region B). Whereas there is no significant difference for phospho-Histone H3, there is a significant increase of TUNEL staining in Actb^−/−^ sections compared to Actb^+/+^ sections. Bars represent mean ± SEM; **P<0.01, ***P<0.001.

To investigate the possibility that less migratory neural crest cells are generated in Actb^−/−^ embryos, we examined apoptosis and proliferation levels by performing TUNEL assays and studying the presence of phospho-Histone H3 on E9.5 Actb^+/+^ and Actb^−/−^ transverse embryo sections using p75 as a neural crest marker. Quantification of TUNEL intensities of pre-migratory neural crest cells in the dorsal neural tube of both Actb^+/+^ and Actb^−/−^ embryos revealed the most significant differences, indicating that Actb^−/−^ neural crest cells exhibit more cell death compared to Actb^+/+^ neural crest cells (Figure 3A,C). Actb^−/−^ migratory neural crest cells also showed slightly higher TUNEL intensities than migratory neural crest cells of Actb^+/+^ embryos, as assessed by quantification of intensities in the region adjacent to the neural tube. However, we could not show differences in proliferation rates between Actb^−/−^ and Actb^+/+^ neural crest cells (Figure 3B,C), indicating that only increased apoptosis accounts (at least in part) for a reduced number of migratory neural crest cells.

**Figure 4:**
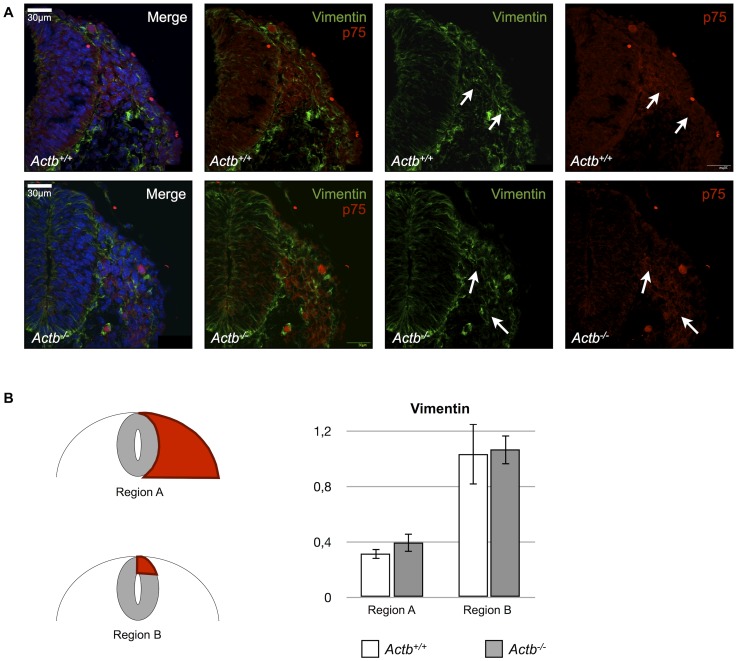
Actb^−/−^ neural crest cells express the mesenchymal marker vimentin in vivo. (A) Vimentin immunohistochemistry on Actb^+/+^ and Actb^−/−^ embryo sections at E9.5. p75 and DAPI were used as markers for neural crest cells and nuclei respectively. Both Actb^+/+^ and Actb^−/−^ sections show comparable levels of vimentin expression in migratory neural crest cells of both regions (indicated by white arrows). (B) Quantification of Vimentin intensities in migratory neural crest cells adjacent to the neural tube (region A) and in the dorsal neural tube (region B). There is no significant difference between Actb^+/+^ and Actb^−/−^ embryo sections. Bars represent mean ± SEM.

Although the Actb^−/−^ neural crest cells show expression of specific neural crest markers, we also examined the presence of vimentin in nascent neural crest, since vimentin is frequently used as a marker for mesenchymal cells or cells undergoing an epithelial-to-mesenchymal transition (EMT) such as delaminating neural crest cells. This marker was indeed expressed in Actb^−/−^ migratory neural crest cells but we could not detect a significant difference between Actb^−/−^ and Actb^+/+^ neural crest cells (Figure 4A,B).

### Actb^−/−^ Neural Crest Cells Display Aberrant Cell Motility ex vivo which is Rescued by Inhibiting Rho-ROCK Signaling

**Figure 5:**
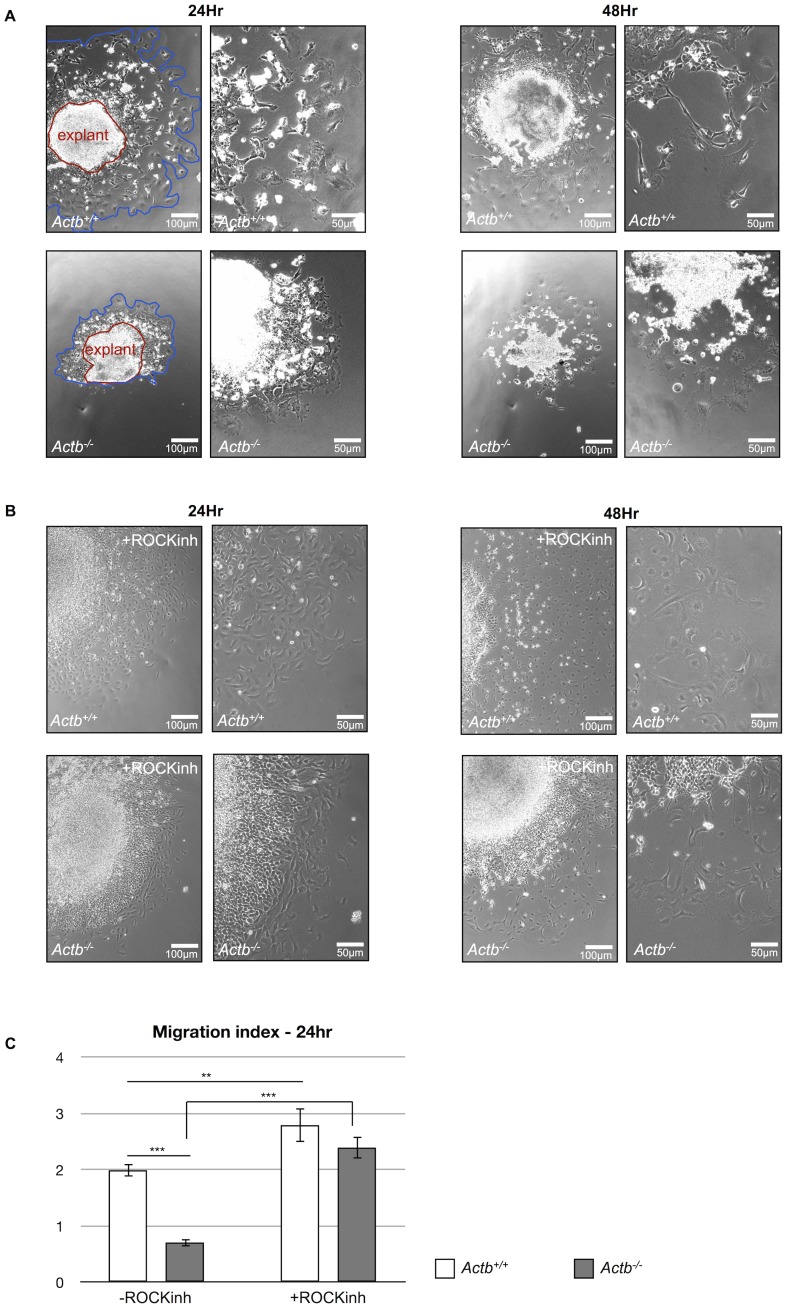
ROCK inhibition rescues impaired migration capacity of Actb^−/−^ neural crest cells ex vivo. (A) Neural tube explant assays reveal a migration defect of Actb^−/−^ neural crest cells ex vivo. After 24 hours the Actb^−/−^ explant (red line) shows a smaller outgrowth area (blue line) than the Actb^+/+^ control neural tube (left panel). After 48 hours, differences between Actb^+/+^ and Actb^−/−^ neural crest cells become more pronounced (right panel). (B) After 24 hours of ROCK inhibition the Actb^−/−^ explant shows a remarkable difference in migration capacity (left panel, compare with 5A left panel). The regained migration capacity of Actb^−/−^ neural crest cells is even more obvious after 48 hours (right panel). (C) Quantification of the neural tube outgrowths. A significant reduction of outgrowth area was found for Actb^−/−^ neural crest cells compared to Actb^+/+^ neural crest cells. When treated with ROCK inhibitor Actb^−/−^ neural crest cells showed increased migration to the level of Actb^+/+^ neural crest cells. Bars represent mean ± SEM; **P<0.01, ***P<0.001.

To document the neural crest migration problem further, we exploited neural crest explants. Neural tubes corresponding to the last formed eight somite pairs were isolated from E9.0 Actb^−/−^ and Actb^+/+^ embryos. After 24 hours, there was an obvious difference in neural crest outgrowth, which became more pronounced after 48 hours (Figure 5A). We quantified the distance (from the initial surface of the explant) of these outgrowths and found significant differences between Actb^−/−^ and Actb^+/+^ neural crest cells resulting in a significantly decreased migration index of Actb^−/−^ neural crest cells (Figure 5C). This suggests that Actb^−/−^ neural crest cells experience migration difficulties.

A migration defect could also be observed in MEFs lacking beta-actin [10], [34]. We showed a role for ROCK in imposing impaired migration of Actb^−/−^ MEFs, since inhibiting the Rho-ROCK signaling pathway could restore their migration capacity [10]. To test if ROCK could also be responsible for the diminished migration of Actb^−/−^ neural crest cells, we inhibited this kinase in neural tube explant assays by adding the cell permeable ROCK inhibitor Y-27632 to the culture medium of the neural crest explants for 48 hours. The results show a drastic improvement in their ability to migrate away from the neural tube (Figure 5B). We detected a significant increase in migration index between Actb^−/−^ neural tubes treated with ROCK inhibitor versus Actb^−/−^ neural tubes without treatment. In addition, we were not able to observe a significant difference between the outgrowths of Actb^+/+^ and Actb^−/−^ neural tubes when both were treated with ROCK inhibitor (Figure 5C). These results confirm a rescue of migration capacity as was also observed for Actb^−/−^ MEFs treated with ROCK inhibitor.

### Aberrant Cadherin Expression in Neural Crest Cells of Actb^−/−^ Embryos

**Figure 6:**
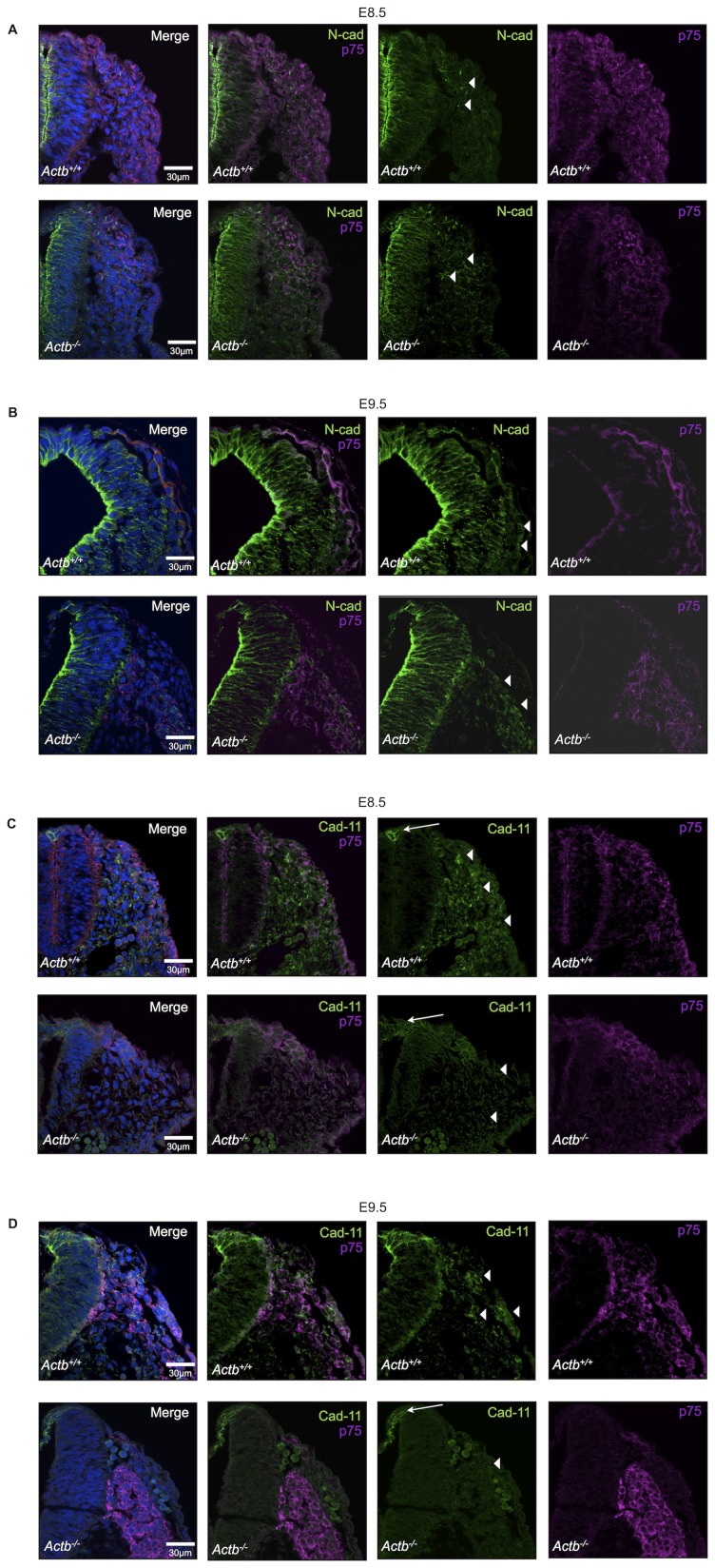
Deregulated cadherin expression in Actb^−/−^ neural crest cells. (A) N-cadherin expression at E8.5 in Actb^+/+^ (upper panel) and Actb^−/−^ (lower panel) embryo sections stained with p75. N-cadherin expression is present in the neural tube and in migrating neural crest cells (white arrowheads), similar in Actb^+/+^ and Actb^−/−^ embryos. (B) N-cadherin expression at E9.5 in Actb^+/+^ (upper panel) and Actb^−/−^ (lower panel) embryo sections stained with p75 in vivo. N-cadherin expression is present in the neural tube and in migrating neural crest cells (white arrowheads), similar in Actb^+/+^ and Actb^−/−^ embryos. (C) Cadherin-11 expression at E8.5 in Actb^+/+^ (upper panel) and Actb^−/−^ (lower panel) embryo sections stained with p75 in vivo. Cadherin-11 expression is present in the dorsal neural tube (white arrow) and in migrating Actb^+/+^ neural crest cells (white arrowheads), similar in Actb^+/+^ and Actb^−/−^ embryos. (D) Cadherin-11 expression at E9.5 in Actb^+/+^ (upper panel) and Actb^−/−^ (lower panel) embryo sections stained with p75 in vivo. Actb^−/−^ neural crest cells show less intense cadherin-11 staining compared to Actb^+/+^ neural crest cells (white arrowheads). In all cases DAPI was used to mark nuclei.

**Figure 7:**
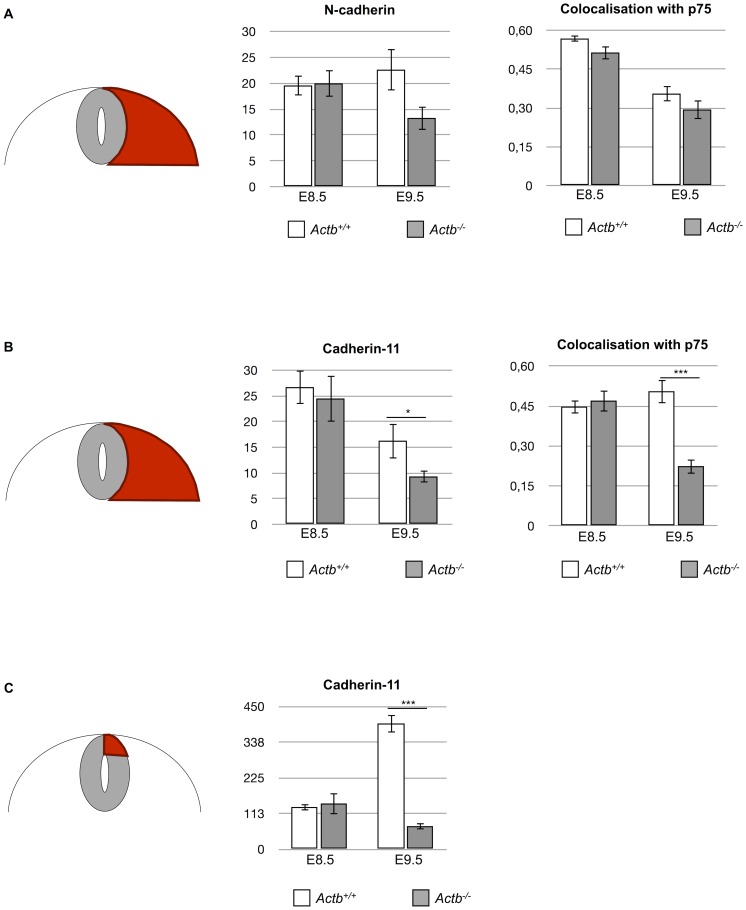
At E9.5 Actb^−/−^ neural crest cells show a significant decrease of cadherin-11 expression. (A) Quantification of N-cadherin intensities in Actb^+/+^ and Actb^−/−^ migratory cells adjacent to the neural tube at E8.5 and E9.5 (left graph, Y-axis: relative intensities) and colocalization of N-cadherin with p75 in migratory cells at E8.5 and E9.5 (right graph, Y-axis: colocalization index on a scale from 0 to 1). Quantification was done on images such as shown in figure 6. No significant differences were observed between Actb^+/+^ and Actb^−/−^ embryos in this region for both stages. (B) Quantification of cadherin-11 intensities in Actb^+/+^ and Actb^−/−^ migratory cells next to the neural tube at E8.5 and E9.5 (left graph, Y-axis: relative intensities) and colocalization of cadherin-11 with p75 in migratory cells at E8.5 and E9.5 (right graph, Y-axis: colocalization index on a scale from 0 to 1). We only observed a significant reduction of cadherin-11 intensities in Actb^−/−^ neural crest cells at E9.5. At the same stage, a significant decrease of cadherin-11 and p75 colocalization was seen as well in Actb^−/−^ neural crest cells. (C) Quantification of cadherin-11 intensities specifically in the dorsal neural tube of Actb^+/+^ and Actb^−/−^ embryos at E8.5 and E9.5. This region also showed a significant reduction of cadherin-11 intensity for Actb^−/−^ embryos. Bars represent mean ± SEM; *P<0.05, ***P<0.001.

The Actb^−/−^ MEFs display altered signaling, in part due to a changed genetic program, including differential expression of adhesion molecules [10]. The nature and the extent of altered signaling in Actb^−/−^ neural crest cells is unknown. It has, however, been well established in other model organisms that cadherins play a significant role in cell sorting, the regulation of tissue formation and in neural crest cell migration [16], [17], [18]. We therefore probed expression of cadherins on sections of embryos at stages E8.5 and E9.5, to investigate if altered cadherin expression plays a role in the observed phenotype. We used p75 as a marker to track the neural crest in combination with different cadherin antibodies: N-cadherin, E-cadherin and cadherin-11. We could not observe E-cadherin expression in the examined regions at E8.5 nor at E9.5 (although other regions of the embryo did stain, data not shown). At E8.5, we did not detect major differences in the expression pattern of both N-cadherin and cadherin-11 between Actb^+/+^ and Actb^−/−^ embryos (Figure 6A,C). N-cadherin is expressed in the whole neural tube of Actb^+/+^ and Actb^−/−^ embryos and remains present in migrating neural crest cells. Cadherin-11 is present in nascent neural crest cells in the dorsal neural tube, and remains strongly expressed in the migratory neural crest, both in Actb^+/+^ and Actb^−/−^ embryos. We quantified the intensity of each cadherin in the migrating neural crest cell population adjacent to the neural tube and the cadherin-11 intensity specifically in the dorsal neural tube and were indeed not able to observe significant differences (Figure 7A–C). In addition, we determined the colocalization of N-cadherin or cadherin-11 with p75 (Figure 7A,B) for migratory neural crest cells using the Pearson’s coefficient and again found no difference between Actb^+/+^ and Actb^−/−^ embryos at this stage.

At E9.5, the Actb^+/+^ expression pattern of N-cadherin remains identical to E8.5 and no gross differences in expression pattern of N-cadherin were observed between Actb^+/+^ and Actb^−/−^ embryos (Figure 6B). Likewise, no significant differences in N-cadherin intensity or colocalization with p75 between both genotypes were observed (Figure 7A). However, at this stage we found drastic differences in the cadherin-11 expression pattern in Actb^−/−^ neural crest cells versus Actb^+/+^ neural crest cells. While Actb^−/−^ neural crest cells are still expressing p75, they mostly lost cadherin-11 expression (Figure 6D), resulting in a significant decrease in colocalization between p75 and cadherin-11 (Figure 7B). Cadherin-11 intensities are reduced in migrating Actb^−/−^ neural crest cells adjacent to the neural tube, and even further decreased in pre-migratory Actb^−/−^ neural crest cells located in the dorsal neural tube (Figure 7B,C). Interestingly, apoptosis of Actb^−/−^ neural crest cells was also highest in pre-migratory neural crest cells, raising the possibility that the loss of cadherin-11 expression correlates with the apoptosis.

With respect to migration, at E8.5 one can appreciate the streams of invading neural crest cells as visualized by the p75 neural crest marker (Figure 6A–D). At this point there is no major difference in the migration patterns between the Actb^+/+^ and Actb^−/−^ embryos. At E9.5, however, most migrating Actb^−/−^ neural crest cells were organized as clusters rather than distinct streams, indicating that well organized migration of neural crest cells is disturbed in Actb^−/−^ embryos after E8.5 (Figure 6A–D).

### Inhibiting the Rho-ROCK Pathway Influences Cadherin-11 Expression, but not N-Cadherin Expression, in Migratory Actb^−/−^ Neural Crest Cells

**Figure 8:**
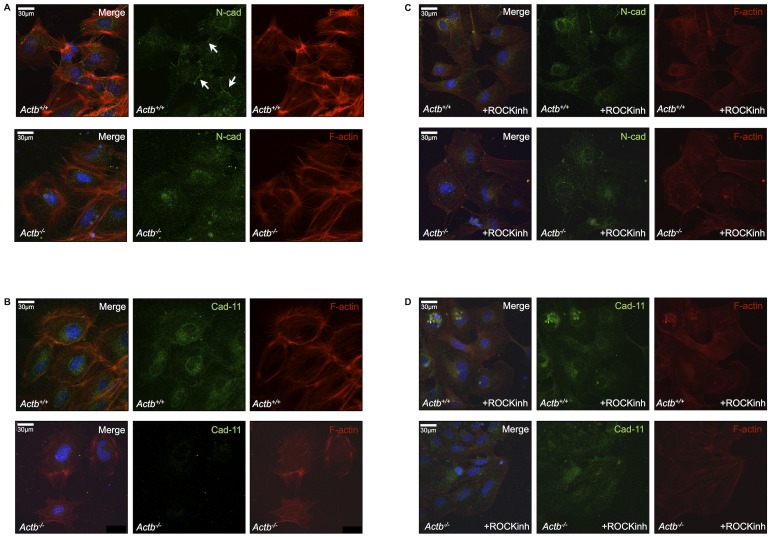
ROCK inhibition affects cadherin expression in Actb^−/−^ neural crest cells ex vivo. (A) N-cadherin expression in Actb^+/+^ (upper panel) and Actb^−/−^ (lower panel) neural crest cells after 48 hours of neural tube culture. Actb^+/+^ and Actb^−/−^ neural crest cells showed comparable N-cadherin expression levels in the cytoplasm. Actb^+/+^, but not Actb^−/−^, neural crest cells display expression of membrane-bound N-cadherin (indicated by white arrows). (B) Cadherin-11 expression in Actb^+/+^ (upper panel) and Actb^−/−^ (lower panel) neural crest cells after 48 hours of neural tube culture. Actb^−/−^ (lower panel) neural crest cells display no cadherin-11 expression compared to Actb^+/+^ (upper panel) neural crest cells. (C) N-cadherin expression in Actb^+/+^ (upper panel) and Actb^−/−^ (lower panel) neural crest cells after 48 hours ROCK treatment of neural tube culture. ROCK inhibition had no effect on N-cadherin expression of Actb^−/−^ neural crest cells. However, membrane-bound N-cadherin was absent from cell-cell contacts of ROCK inhibitor treated Actb^+/+^ neural crest cells. (D) Cadherin-11 expression in Actb^+/+^ (upper panel) and Actb^−/−^ (lower panel) neural crest cells after 48 hours ROCK treatment of neural tube culture. Expression of cadherin-11 in Actb^−/−^ neural crest cells (lower panel) was increased after ROCK treatment relative to non-treated cells. Note that for both Actb^+/+^ and Actb^−/−^ neural crest cells ROCK inhibition resulted in the expected disruption of stress fibers. Imaging of fluorescence was done at similar laser settings.

Since we observed diminished cadherin-11 expression in Actb^−/−^ neural crest cells in the embryo (Figure 7B–C) and treatment with ROCK inhibitor could rescue the migration capacity of these neural crest cells (Figure 5B–C), we examined whether inhibiting Rho-ROCK signaling had effects on cadherin expression ex vitro. We first investigated N-cadherin and cadherin-11 expression in migrating neural crest cells using neural tube explants from E9.0 embryos. Actb^+/+^ neural crest cells showed expression of membrane-bound N-cadherin at regions of cell-cell contacts (indicated with arrows) and in the cytoplasm. In Actb^−/−^ neural crest cells, we only observed cytoplasmic N-cadherin expression and thus no membrane-bound forms were detectable (Figure 8A). Similar to what we observed in vivo, we did not detect cadherin-11 expression in migratory Actb^−/−^ neural crest cells compared to Actb^+/+^ neural crest cells that display cytoplasmic expression of this cadherin (Figure 8B).

We subsequently inhibited ROCK by adding 10 µM of the ROCK inhibitor Y-27632 to the culture medium of the neural crest explants for 48 hours and monitored occurrence and subcellular localization of the two investigated cadherins. After treatment with ROCK inhibitor, N-cadherin expression in the cytoplasm was comparable for Actb^−/−^ neural crest cells versus Actb^+/+^ neural crest cells (Figure 8C). Interestingly, Actb^+/+^ neural crest cells showed a downregulation of membrane-bound N-cadherin at regions of cell-cell contact, confirming previous observations in quail [35]. By contrast, ROCK inhibition improved cadherin-11 expression in Actb^−/−^ neural crest cells, indicating that the increased migration capacity is accompanied by an increase of cadherin-11 expression in Actb^−/−^ cells (Figure 8D).

## Discussion

Our results reveal a role for beta-actin during neural crest ontogeny. Whereas it is increasingly appreciated that deregulation of neural crest cell migration is associated with disease states [36], [37], [38], our understanding of early neural crest ontogeny in mouse is not complete [39], [40]. In addition, conflicting observations exist between mouse and other species such as chick and quail [19], [35], [41], [42], [43]. In our study, we detected that Actb^−/−^ neural crest cells fail to populate distant sites in the embryo. We showed that this is partly due to the observation that pre-migratory Actb^−/−^ neural crest cells display increased apoptosis and partly due to impaired migration including aberrant clustering of migratory neural crest cells. At the molecular level the Actb^−/−^ neural crest population displayed decreased cadherin-11 expression which was especially the case for the pre-migratory neural crest population.

### Is Beta-actin a Modulator of Cadherin-11 Expression Influencing Neural Crest Cell Survival?

The migration defect can easily be accepted given the role of beta-actin in cell migration in other cellular systems [10], [34], [44]. However, the affected cadherin signaling and apoptosis are more surprising. Decreased cadherin-11 expression and increased apoptosis are both more pronounced in the pre-migratory neural crest cells population and less pronounced in the migratory neural crest cell population. The requirement of cadherin-11 for migration is consistent with a number of studies using different cell types such as neural precursors, neural crest cells and smooth muscle cells [45], [46], [47]. Also the survival function of cadherin-11 was already described in literature [48]. It remains however to be investigated whether the two observations, which show a remarkable overlap in timing and location, are mechanistically linked. Even more puzzling is the connection with beta-actin in neural crest cells. In the few studies that used beta-actin ablation in mouse models, apoptosis was never studied. However, we recently demonstrated a role for beta-actin in cell homeostasis and as a transcriptional modulator of Gata2 during mouse embryogenesis [10], [12]. Nuclear functions of actin have been contested for more than 20 years but a role for actin as a transcriptional modulator is now firmly established. However, to date only a handful of manuscripts describe a link between a particular actin isoform and specific genes. We speculate that Gata2 is not the only gene which expression is partially controlled by beta-actin. We base this hypothesis on previous results from the Actb^−/−^ MEFs showing a drastic genetic reprogramming of many genes, including cadherins [10]. We therefore hypothesize that, in the neural crest cells studied here, this is the case as well although it remains to be investigated whether the altered cadherin-11 expression is a direct or an indirect effect of beta-actin ablation. The observation that by inhibiting the Rho kinase ROCK we not only restored the impaired migration behavior of Actb^−/−^ neural crest cells, but also ameliorated cadherin-11 expression further suggests actin cytoskeletal control of cadherin-11 expression.

### Beta-actin is Important in Early Phases of Nervous System Development

In previous work, we and others reported impaired migration of Actb^−/−^ MEFs or T-cells [10], [34]. We here demonstrate migration defects upon ablation of beta-actin in another cell type: neural crest cells. In vivo this leads to a striking absence of a well formed peripheral nervous system, as evidenced by lack of neurofilament and beta-III tubulin staining, suggesting an important function for beta-actin in peripheral nervous system development. This contrasts studies where conditional ablation of beta-actin is not required for motor neuron function or regeneration in vivo [49] or resulted in restricted morphological abnormalities in the central nervous system [9]. This however does not contradict our results since a complete downregulation of beta-actin during the early phases of development was never achieved with the conditional ablation setup.

### A Role for Cadherin-11 in Mouse Trunk Neural Crest Migration

**Figure 9:**
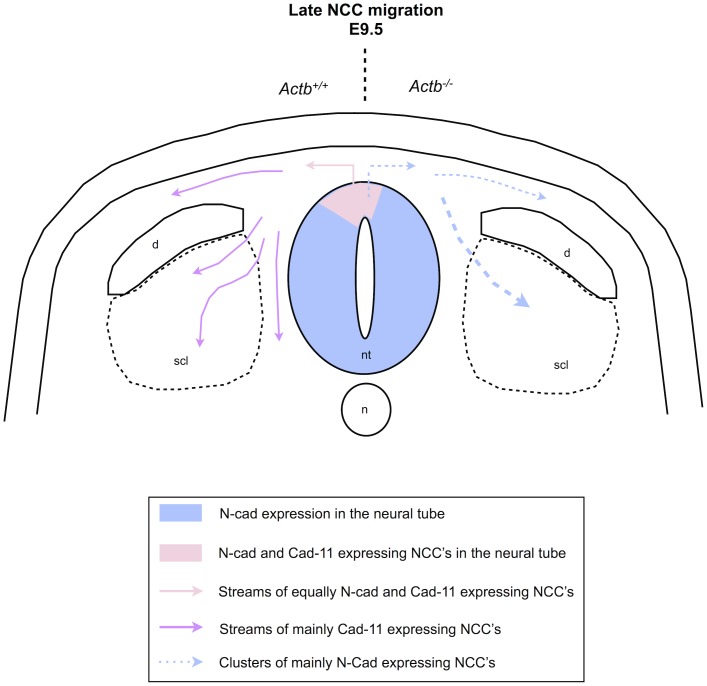
Actb^−/−^ neural crest cells display decreased cadherin-11 expression and migrate in clusters. Schematic view of N-cadherin and cadherin-11 expression in the Actb^+/+^ and Actb^−/−^ embryos at E9.5. Neural crest cells in the dorsal neural tube express both N-cadherin and cadherin-11, migrating neural crest cells express high levels of cadherin-11 while maintaining N-cadherin levels. Actb^−/−^ neural crest cells in the dorsal neural tube show very low levels of cadherin-11. Migrating Actb^−/−^ neural crest cells show decreased cadherin-11 expression levels and therefore mainly express N-cadherin. Most migrating Actb^−/−^ neural crest cells were organized as clusters rather than distinct streams as Actb^+/+^ neural crest cells. n: notochord; nt: neural tube; d: dermomyotome; scl: sclerotome.

Cadherin expression during neural crest ontogeny is well studied in chick and Xenopus, however, details of cadherin expression during mouse neural crest ontogeny are lagging behind [19], [48], [50], [51], [52], [53], [54]. Cadherin-11 expression patterns in mouse cranial neural crest cells were previously investigated [55]. Cadherin-11 was localized in most mesenchymal cells in the head region and especially neural crest cells constituting the mandibular and maxillary arches expressed high levels. The developing cranial neural folds also expressed this cadherin. These authors additionally reported cadherin-11 expression in the dorsal midline of the neural tube in the posterior trunk region. This most likely matches the cadherin-11 expression pattern that we observed in the trunk dorsal neural tube of Actb^+/+^ embryos, corresponding to the newly formed pre-migratory neural crest population. We can conclude that the cadherin-11 expression pattern during the early phases of neural crest development is very similar in cranial and trunk regions of mouse embryos (Figure 9, left part). Initial expression is seen in the pre-migratory neural crest territory: neural fold ridges in the cranial region and the dorsal part of the neural tube in the trunk region. Afterwards, expression of cadherin-11 is retained in the migratory cell population, both in the cranial regions as the trunk regions. Similar cadherin-11 expression was observed during rat development [56].

Ablation of beta-actin leads to decreased cadherin-11 expression in the neural crest population, specifically in the pre-migratory neural crest cell population, and to impaired migration behavior. Figure 9 shows a schematic view of the E9.5 neural crest migration pattern in Actb^+/+^ and Actb^−/−^ embryos in relation to cadherin expression. Whereas Actb^+/+^ neural crest cells in the dorsal neural tube expressed both cadherin-11 and N-cadherin, Actb^−/−^ neural crest cells in the dorsal neural tube mostly lost their cadherin-11 expression. Emigrating Actb^−/−^ neural crest cells therefore mainly express N-cadherin instead of the combined cadherin-11 and N-cadherin expression that emigrating Actb^+/+^ neural crest cells display. Whereas in Actb^+/+^ embryos neural crest cells migrate as distinct streams of mainly cadherin-11 expressing cells, in Actb^−/−^ embryos they rather migrate as large clusters which show decreased cadherin-11 expression. In addition, the neural crest cells show increased apoptosis, especially in the pre-migratory population, similar to the decreased cadherin-11 staining.

At this point, it is difficult to say which events are cause and which are consequence. Based on our previous results [10], [12] we assume that ablation of beta-actin causes altered cadherin expression, via genetic reprogramming or as part of a transcriptional complex instructing cadherin-11 transcription. Data from many different cell types support the hypothesis that decreased cadherin-11 signaling could elicit impaired migration [45], [47], [48], [53], [57], [58], [59], [60], [61]. Cadherin-11 is a type II cadherin and indeed considered as a marker of mesenchymal, migratory phenotypes [55], [56], [62]. On the other hand, altered cadherin expression in combination with apoptosis has frequently been reported in many different tissues as well [63], [64], [65], [66].

### Actb^−/−^ Neural Crest Cells Lack Membrane-bound N-cadherin

We never detected plasma membrane-bound N-cadherin in Actb^−/−^ neural crest cells, suggesting that beta-actin is necessary for the membrane-bound localization of this cadherin. N-cadherin was demonstrated to have important polarizing functions during neurulation and development of neurons [71], [72], [73], [74], [75]. Along similar lines it was recently reported that beta-actin is required for establishing apicobasal cell polarity in intestinal epithelial cells [76]. Moreover beta-actin seems important for the polarization of fibroblasts via cadherin-11 [59]. Cells expressing a defective variant of cadherin-11 revealed a more extensive cortical F-actin ring that correlated with significant higher levels of activated Rac1 and resulted in impaired intercellular motility and problems to rearrange in multicellular clusters. Also in neural crest cells an important balance of Rac is necessary to define the front and rear of the cell [77]. We hypothesize a similar scenario could be at play in these Actb^−/−^ neural crest cells via alternation of N-cadherin expression due to beta-actin ablation.

A phenomenon which appears to be common to many species is the exchange of a type I cadherin for a type II cadherin prior to major mophological movement [67], [68], [69], such as neural crest emigration [21], [50], [70]. We here demonstrate that emigrated neural crest cells retain N-cadherin expression, at least in the early stages of migration, suggesting the cadherin switch in mouse trunk is more subtle than previously described for other species such as chick and quail [55], [68], [69].

### Deregulated ROCK Activity Contributes to the Cellular Phenotypes

We previously demonstrated that Actb^−/−^ MEFs were able to migrate in the absence of beta-actin if ROCK was inhibited [10]. This is recapitulated in this study and thus a similar scenario seems to apply to Actb^−/−^ neural crest cells isolated from embryos. Several published observations support a regulatory role for ROCK in neural crest ontogeny [35], [43]. Phillips et al. used mice expressing a dominant negative construct of ROCK (ROCKDN) in the neural crest lineage [43]. Similar to our Actb^−/−^ embryos, control and ROCKDN embryos were indistinguishable based on their external appearance at E8.5. At later stages, these mice exhibited neural crest deficiency and hypoplastic neural crest derived craniofacial structures, suggesting that ROCK activity is essential for neural crest cells to form the craniofacial region. The extent of altered ROCK activity in the Actb^−/−^ neural crest cells is however currently unknown (due to technical difficulties to measure this) but is probably increased based on the effect of the inhibitor and on observations of the Actb^−/−^ MEFs [10]. Also in quail, a negative modulatory role for Rho and ROCK signaling in delamination of neural crest cells has been reported [35]. The inhibition of Rho-ROCK promotes premature and enhanced neural crest delamination, concomitant with the disruption of the F-actin cytoskeleton. The authors also reported an interaction between N-cadherin and Rho-ROCK via regulation of F-actin dynamics in quail neural crest cells. N-cadherin expression is normally lost from delaminating cells due to intracellular cleavage which generates a C-terminal fragment (CTF) [35], [78]. However, inhibition of ROCK results in neural crest cells devoid of N-cadherin and losing intercellular adhesions prematurely. Our results also indicate a connection between ROCK and cadherins since ROCK inhibition eliminated N-cadherin from the membrane of wild type neural crest cells. Conversely, treatment of Actb^−/−^ neural crest cells with ROCK inhibitor not only rescued their migration potential but also let to an increase in cadherin-11 expression in these cells, potentially positioning cadherin-11 in a feedback loop with Rho-ROCK signaling. A more direct connection between a cadherin and Rho was already described in Xenopus, where it was shown that cadherin-11 acts upstream of RhoA [48].

In summary, we revealed a novel role for beta-actin during development. Absence of beta-actin severely hampers formation of the peripheral nervous system, due to defective neural crest migration. At the cellular level Actb^−/−^ neural crest cells show increased apoptosis, lack cadherin-11 expression and membrane-bound N-cadherin. Treatment with ROCK inhibitor not only rescued the migration capacity of Actb^−/−^ neural crest cells but also increased cadherin-11 expression. This suggests there might be a reciprocal connection between Rho-ROCK signaling and cadherin-11, since the influence of cadherins on this pathway in neural crest cells was already reported [35], [48], [79]. In addition, the absence of membrane-bound N-cadherin in Actb^−/−^ neural crest cells suggests a role for beta-actin in the polarization of neural crest cells.
